# Conflict of interest disclosure in biomedical research: a review of current practices, biases, and the role of public registries in improving transparency

**DOI:** 10.1186/s41073-016-0006-7

**Published:** 2016-05-03

**Authors:** Adam G. Dunn, Enrico Coiera, Kenneth D. Mandl, Florence T. Bourgeois

**Affiliations:** 1 Centre for Health Informatics, Australian Institute of Health Innovation, Macquarie University, Sydney, NSW 2109 Australia; 2 Department of Pediatrics, Harvard Medical School, Boston, MA 02115 USA; 3 Computational Health Informatics Program, Boston Children’s Hospital, Boston, MA 02115 USA; 4 Department of Biomedical Informatics, Harvard Medical School, Boston, MA 02115 USA

**Keywords:** Journal Editor, Accessible Registry, Financial Conflict, Physician Payment, Prospective Registration

## Abstract

Conflicts of interest held by researchers remain a focus of attention in clinical research. Biases related to these relationships have the potential to directly impact the quality of healthcare by influencing decision-making, yet conflicts of interest remain underreported, inconsistently described, and difficult to access. Initiatives aimed at improving the disclosure of researcher conflicts of interest are still in their infancy but represent a vital reform that must be addressed before potential biases associated with conflicts of interest can be mitigated and trust in the impartiality of clinical evidence restored. In this review, we examine the prevalence of conflicts of interest, evidence of the effects that disclosed and undisclosed conflicts of interest have had on the reporting of clinical evidence, and the emerging approaches for improving the completeness and consistency of disclosures. Through this review of emerging technologies, we recognize a growing interest in publicly accessible registries for researcher conflicts of interest and propose five desiderata aimed at maximizing the value of such registries: mandates for ensuring that researchers keep their records up to date; transparent records that are made available to the public; interoperability to allow researchers, bibliographic databases, and institutions to interact with the registry; a consistent taxonomy for describing different classes of conflicts of interest; and the ability to automatically generate conflicts of interest statements for use in published articles.

## Background

For researchers, conflicts of interest describe situations where the impartiality of research may be compromised because the researcher stands to profit in some way from the conclusions they draw [1]. The clearest and most often discussed example of a conflict of interest in biomedical research involves doing research on a specific intervention while receiving research funding or personal remuneration from the company producing that intervention. While there are many other forms of financial and non-financial conflicts of interests [2], this is the type that is most often measured and discussed. In practice, every researcher holds a set of interests—financial, personal, ideological, or otherwise—which may lead to bias in the context of specific research. The topic of disclosing conflicts of interest has been debated since the 1980s [3], with disagreements about whether or not conflicts of interest should be disclosed and whether methods of peer review are sufficient for mitigating the potential for bias associated with research undertaken by researchers who hold conflicts of interest.

Despite a general consensus favoring disclosure, and nearly 20 years after disclosures have been required for submissions to medical journals [4], conflicts of interest are still often missing from published articles, guidelines, and news media [5–8]. The lack of transparency in the disclosure of conflicts of interest is a problem in biomedical research because it hinders our ability to mitigate the risk of bias. These biases, when hidden, can affect clinical decision-making by making interventions appear safer or more effective than they really are. High-profile examples where undisclosed conflicts of interest have clearly affected clinical practice may have contributed to the erosion of public trust in biomedical research and peer review processes [9–14]. New methods for further improving the completeness and consistency with which researchers disclose their conflicts of interest are now needed to support mitigation and increase trust in peer-reviewed research. In this review, we provide a narrative review of studies that have measured the prevalence of disclosed and undisclosed conflicts of interest, summarize what is and what is not known about associations between conflicts of interest and biased reporting, describe some of the pertinent examples of where conflicts of interest appear to have affected the presentation of clinical evidence or public opinion, and discuss some recent and emerging approaches aimed at improving the accuracy and completeness of disclosures [6, 15, 16]. We conclude by speculating on the benefits of a global, publicly accessible registry for recording researchers’ declarations of interests and recommend five key features that would maximize its value in measuring risk of bias due to conflicts of interest.

### Conflicts of interest are common and underreported

Studies measuring the incidence of disclosed and undisclosed conflicts of interest have to date been generally small, heterogeneous in terms of setting and outcomes, and often focused largely on the subset of conflicts of interest that are financial in nature. In a 2003 systematic review of the prevalence of conflicts of interest, Bekelman et al. [1] found that a third of biomedical researchers in academic institutions have held conflicts of interest that could introduce a risk of bias. Cross-sectional studies across a heterogeneous set of conditions suggest that between 29 and 69 % of published clinical trial reports include disclosures of conflicts of interest [17–24].

Studies measuring undisclosed conflicts of interest suggest that between 43 and 69 % of study reports and other articles fail to include disclosures of conflicts of interest [5, 6, 25, 26]. However, these values are not directly comparable because the studies involve different types of conflicts of interests and data sources and are restricted to specific financial conflicts of interests and the study sizes are relatively small. In a cross-sectional study of published clinical practice guidelines issued by medical organizations in the USA and Canada, 48 % of authors disclosed conflicts of interest and 25 % formally declared none, among which 11 % were found to have undisclosed conflicts of interest [7]. In a study of Danish clinical practice guidelines, 96 % of guidelines included at least one author with a conflict of interest, but only 2 % of the guidelines disclosed those conflicts of interest [27].

Conflicts of interest appear to be even more rarely reported in science journalism. A 2007 study showed that very few newspaper stories about scientific research report the financial ties of researchers and quoted sources, even when the conflicts of interest are disclosed in the journal article [8]. In 2013, Mandeville et al. [28] found that 3 of 425 newspaper articles on the 2009 to 2010 A/H1N1 pandemic noted the competing interests of the quoted researchers.

### Conflicts of interest can introduce biases that lead to harm

While we know of no systematic review that quantifies the risk of bias associated with the presence of financial and non-financial conflicts of interest, a group of observational studies have shown that financial conflicts of interests are associated with biases across the spectrum of biomedical research. Researchers with conflicts of interest were found to be more likely to choose comparators that would produce favorable results [29], selectively include only certain outcomes in published reports [30], publish conclusions that are inconsistent with the study results [31, 32], or complete a clinical trial without subsequent publication of the results [33]. These types of biases can also impact the quality and reliability of systematic reviews, arguably the most critical publications guiding clinical care [34, 35]. When authors of systematic reviews hold financial conflicts of interest, they are more likely to interpret data as evidence supporting an intervention [9, 36, 37]. Contributors to clinical practice guidelines are more likely to recommend the intervention in clinical practice if they hold a conflict of interest [10, 38, 39].

Both disclosed and undisclosed conflicts of interest can have a negative impact on clinical evidence, public opinion, and clinical decision-making [40]. For example, following a meta-analysis linking rosiglitazone to an increased risk of myocardial infarction, researchers with conflicts of interest continued to defend the drug, often failing to disclose links to pharmaceutical companies [11], and this may have further delayed the market withdrawal of the drug in several countries. Table 1 enumerates some examples of the influence of undisclosed researcher conflicts of interest on patient care through published research reports and statements in the news media.

**Table 1:** Examples of the impact of undisclosed conflicts of interest on clinical evidence, public opinion, and clinical decision-making

Interventions	The potential impact of undisclosed conflicts of interest
Rosiglitazone	Following a meta-analysis showing an association between rosiglitazone and cardiovascular risk, articles authored by researchers with conflicts of interest were more likely to uphold the safety of the drug [11]. Among the articles with identified conflicts of interest, 23 % did not disclose them. Rosiglitazone was withdrawn from the market for safety reasons in several countries but remains available in the USA.
Alteplase	Alteplase was strongly recommended for use in acute stroke in clinical guidelines despite resistance from emergency physicians concerned about intracerebral hemorrhage [84]. Seven of eight panelists developing the guidelines had potential conflicts of interest (indirect financial ties to the manufacturer of alteplase), but only three of the panelists disclosed these conflicts [85]. After the conflicts of interest were revealed, the American Heart Foundation withdrew statements that the intervention could save lives [13].
Risperidone	While failing to completely disclose financial relationships with the manufacturer of risperidone, an influential researcher was instrumental in expanding the diagnosis criteria for bipolar disorder in children and conducted a number of pediatric clinical trials demonstrating the benefit of the drug in children [86]. A congressional investigation later found him guilty of violating federal and university regulations and conflicts of interest policies.
Calcium-channel antagonists	A survey study found that authors’ published positions on the safety of calcium channel antagonists were more likely to be favorable to the drug class if they responded that they had a financial conflict of interest (63 % of authors reported a financial conflict of interest in the survey) [12]. However, only 2 of the 70 articles authored by the respondents included disclosures.
Measles, mumps, rubella (MMR) vaccine	A study linking the MMR vaccine to autism was eventually retracted after it was discovered that an author failed to disclose how he stood to gain financially by discrediting the vaccine [87, 88]. The impact on vaccine decision-making persists even a decade later, with surveys showing that more than one in five people believe that vaccines cause autism [89].
Neuraminidase inhibitors	Academics who were interviewed in newspaper articles covering the 2009 H1N1 pandemic were more likely to overestimate the risk of the pandemic or promote the use of neuraminidase inhibitors if they had conflicts of interest [28]. Only 3 of 425 newspaper articles noted the academics’ conflicts of interest.

### Unintended consequences of disclosing conflicts of interest

Concerns have been raised about the possibility of unintended consequences and burdens associated with disclosing conflicts of interest [41]. For researchers, the disclosure of conflicts of interest may exacerbate biases in the presentation of research by creating the impetus to compensate for the disclosure [42–45]. For readers, the presence of a disclosed conflict of interest may influence their response to information in unexpected ways, including increasing their trust or making them dismissive of the material [46, 47]. However, in a recent study within a small group, it was found that the disclosure of a financial conflict of interest had little impact on a lay audience’s interpretation of research and helped to moderate the concerns of audiences with training in ethics [48].

Journal editors have implemented different policies for addressing researcher conflicts of interest, with some outright avoiding publication of certain article types submitted by authors with industry relationships [49]. In a series of editorials, one journal recently questioned the division this may have created between industry and academia [50–53], which garnered passionate and wide-ranging responses from previous and current journal editors [54, 55].

Regardless of journal policy in the area, nearly half of all clinical trials completed each year are partially or completely funded by industry [56], indicating that a substantial proportion of primary clinical evidence is being produced by researchers who hold conflicts of interest. Industry-sponsored studies differ from otherwise-funded studies across several key characteristics in terms of their designs. Individual studies on specific conditions and interventions have found that industry-sponsored studies enroll larger numbers of patients tend to have lower risk of bias in relation to blinding, may select different comparators, and may select different measurable outcomes [29, 34, 57, 58]. Given the current reliance on industry funding for the production of primary evidence, a blanket dismissal or exclusion of industry authors across all article types is too simplistic, would greatly reduce the volume of evidence available for many interventions, and is unlikely to improve the quality of clinical evidence for most interventions.

### The limits of disclosure

In previous reviews, researchers have concluded that disclosure alone is not enough to mitigate the effects of researchers’ conflicts of interest [1, 59]. To paraphrase Thompson [60], disclosure only reveals the possibility of bias, without providing any guidance for resolving it.

Improved disclosure alone is unlikely to solve the problem of biases associated with conflicts of interest. Rather, improvements in the disclosure of conflicts of interest should be viewed as a way of providing increasingly precise observations of one factor that can influence the design and reporting of research. Given that biases introduced by conflicts of interest are not adequately explained by the differences measured using standard risk-of-bias tools [34, 35], it makes sense to treat conflicts of interest in the same way as we treat any other confounder that may affect the results of a study. The more precisely we can measure the association between different classes of conflicts of interest and biases in design or reporting, the more effectively we can statistically account for them when synthesizing evidence.

### Methods for improving the disclosure of potential conflicts of interest

Systems have been put in place with the aim of measuring or mitigating the biases associated with conflicts of interest for researchers and clinicians. These changes include the introduction of clinical trial registries [61–64], and the release of information related to payments from pharmaceutical companies to physicians [65]. Together with associated policy changes, these systems have been successful in revealing the biases associated with conflicts of interest but it is unclear whether these systems have helped to mitigate these biases.

The databases of registered clinical trials are used to measure and mitigate reporting biases [64, 66]. Lessons about how to introduce a registry for detailing researchers’ declared interests can be gleaned from the successful implementation of ClinicalTrials.gov, which has helped to make the prospective registration of clinical trials a standard practice. The registry was first implemented in 2000 following the Food and Drug Administration Modernization Act of 1997 with reporting requirements further expanded in the Food and Drug Administration Amendments Act of 2007. The registry was endorsed by the International Committee of Medical Journal Editors (ICMJE) which, in 2005, established a policy mandating the prospective registration of clinical trials as a prerequisite for publication in member journals [67]. This action along with support by other journals and federal policies greatly accelerated rates of compliance with trial registration [63], although compliance with registration and results reporting is not yet perfect [30, 68]. For instance, journals still publish trials that were not prospectively registered [69, 70], even where it is a precondition of publication [30], and inconsistencies between information in registries and manuscripts do not appear to influence publication [71]. Today, the registry is the largest single database of clinical trials conducted in the USA and internationally, and its growth highlights the importance of providing not only the tools for reporting and sharing information but also encouraging uptake through changes in policy. When clinical trial publications fail to completely report measurable outcomes, or remain unpublished after an extended delay, clinical trial registries can be used to audit these biases [72], and may help to identify unpublished evidence.

Another example that can be used to inform the development of a publicly accessible registry for declarations of interests comes from recent changes that require physicians in the USA to publicly document relationships between physicians and pharmaceutical industry. The USA passed the Physician Payments Sunshine Act in 2007, which sought public reporting of all financial ties between physicians and pharmaceutical companies. This was followed in 2014 by Open Payments—the release of individual and identifiable financial conflict of interest information for individual physicians by the United States Centers for Medicare and Medicaid Services. This information was publicly released in a searchable database that includes reports on payments to approximately 546,000 licensed physicians in the USA. In 2011, a unique identifier for researchers was proposed for linking physician payments to authors of biomedical research [73]. Some concerns have been raised about the unintended consequences of publicly releasing payment information, including unexpected patient reactions, deliberate underreporting, and payments to non-physicians [74].

### Moving to a publicly accessible registry for disclosing interests

There are two main locations where researchers’ declared interests are currently stored, but these provide little value because they are highly fragmented and largely inaccessible to the public. The first major source of information about conflicts of interest is published articles—but conflicts of interests disclosed in articles are inconsistently described, only include interests that are judged as relevant in the context of the research reported in the article, not uniformly presented in the same places in the article’s text or metadata, and often not amenable to web crawling due to constraints from subscription paywalls and terms of use. The other major location where information about researchers’ interests are stored includes the medical institutions, funding bodies, and biomedical companies that maintain internal registries of conflicts of interest or records of transactions that might constitute conflicts of interest for the researchers they employ or support—but these records are almost invariably private.

In 2012, an Institute of Medicine (IOM) initiative described a centralized repository for conflicts of interest disclosures and highlighted several advantages including a reduced burden for researchers and organizations, the ability to harmonize on standardized definitions, and the ability to quickly capture discrepancies when including multiple sources [15]. In mid-2015, the Association of American Medical Colleges developed *Convey* based on the IOM recommendations and sought to become a central repository for researchers to store records of their financial conflicts of interest. In 2014, Rasmussen et al. [6] advocated for the use of public information from registries where physicians and drug companies are responsible for providing information about industry relationships. Other examples of emerging registries include a voluntary register aimed at doctors registered in the UK with just over 250 entries [16] and reported plans to extract and aggregate disclosures from existing published articles [75].

To populate a centralized registry for conflicts of interest, we recommend the development of computational methods for accessing and aggregating information from published articles and the linking of local private sources of information with the public registry. We propose five desiderata to ensure the growth of the registry and its ongoing comprehensiveness once it has been populated with existing records (Table 2).

**Table 2:** Five key features of a global public registry for researcher conflicts of interest

Key feature	Description
Enforceability	Mandates from publishers, funding bodies, and institutions to require up-to-date details in the registry prior to publication, funding, or as a condition of employment
Transparency	A transparent, archived record of changes, including information on the timing and authorship of entry modifications
Interoperability	The ability for institutions, companies, and other organizations to push changes into the registry and automatically update researcher records
Taxonomy	A consistent taxonomy for describing financial and non-financial conflicts of interest
Automated disclosures	The ability to automatically generate statements about relevant disclosures for inclusion in abstracts and published articles according to templates specified by individual journals

#### Enforceability

For a global public registry of researchers’ declared interests to be comprehensive, it requires a critical mass of support from researchers, journal editors, institutions, companies, and funding organizations. When ClinicalTrials.gov was launched in 2000, its success was not immediate. Recommendations in 2003 suggested that for the clinical trial registry to be comprehensive, it would require support from the National Institutes of Health, industry leaders, journal editors, and lawmakers [64]. More recently, there has been a strong push for the release of patient-level trial data [76–78], with buy-in from both sides of the industry divide and indications that access to some types of patient level data is on the horizon. We are still lacking a corresponding level of unified pressure to ensure the comprehensive and transparent reporting of conflicts of interest disclosures.

#### Transparency

A public record of changes to the registry would ensure that conflicts of interest disclosures can be audited, promoting the accuracy of the recorded information. The registry should permit the editing of records by individual researchers and permanently store public listings of the history of changes for all entries in the registry. In relation to the updating of registry information and the currency of the information available, the ICMJE and individual institutions could lead the development of publishing standards by requiring authors to update their registry profile as part of the manuscript submission or publication process.

#### Interoperability

Academic institutions, funding organizations, and external organizations could link internal registries to the public registry to update researcher information as they secure new funding, commercialize products or services, or enter into new agreements with companies and other organizations. To enable this form of interoperability, the registry must uniquely identify each active researcher. Where previous registries for disclosing industry relationships relied on uniquely identifying practicing physicians and doctors within individual countries [16, 73], it is much harder to reconcile the set of active researchers globally, and this may hinder the coverage of a registry. However, unique identifiers for researchers already exist to track authors across publications. The Open Researcher and Contributor ID (ORCID) is one candidate for the registry. It assigns unique identifiers to authors, allows users to update their own records, and supports the system-to-system communication that would be needed to link disclosed conflicts of interest to researchers and their publications. Another option is PubMed from the US National Library of Medicine, which assigns unique identifiers to authors, and includes programmatic access. However, the facilities provided by the NLM for authors to update, merge, or disambiguate their own record of publication are not widely used outside of the USA. Other systems like Google Scholar profiles provide author identifiers and the ability to disambiguate and remove articles, but do not yet provide public access for the system-to-system communication that would be required.

#### A taxonomy of disclosures

In order to clearly distinguish between the variety of financial and non-financial interests of a researcher and the specific conditions and interventions with which these interests may be in conflict, the registry should use an agreed and consistent taxonomy. A standardized language for describing conflicts of interest would enable users and researchers to better understand the potential impact of various types of conflicts of interest—in particular for non-financial conflicts of interest, for which there is a lack of evidence about prevalence and impact. In 2010, Rochon et al. [79] produced a checklist for clinical research studies that includes structured information appropriate for clinical trials, and this might provide partial information for primary studies. The IOM report commented on the difficulty of producing a standardized format for reporting disclosures [15], suggesting that this remains an unsolved problem in the area.

#### Automated disclosure

Registry data could be particularly useful to journal editors and the lay press if an Application Programming Interface (API) is developed to allow users to read and write conflicts of interest information for researchers using their unique identifiers. Publishers could support journals by using the API to create customizable templates that are automatically filled with authors’ relevant disclosures during the publishing process. Methods for tagging researchers’ interests by condition, intervention, or other medical concept may permit the automatic identification of conflicts. For example, methods applied in information retrieval use medical concept similarity to identify relationships between documents [80–82], which could be adapted to suit this purpose. These would work by matching the set of tags attached to each item in a researcher’s list of potential conflicts of interest with the interventions studied in a trial being submitted to a journal for review, assisting or replacing the manual identification of the subset of relevant disclosures.

### Implications

If implemented to meet each of the above desiderata, a global public registry for researcher conflicts of interest would benefit multiple stakeholders in the research enterprise. The disclosure process would be more efficient for researchers, who could rely on a central repository of comprehensive, up-to-date, and standardized conflict of interest information for inclusion in research reports. Journal editors and peer reviewers could query the database to assess the types of conflicts of interest held by potential reviewers [83]. Clinicians could evaluate research reports in the context of clear and concise descriptions of conflicts of interest. Similarly, journalists could support their readers in understanding and gauging research findings by providing relevant links in media reports to the registry.

To be clear, the act of disclosing a set of interests that could be in conflict with a researcher’s work does not alone guarantee that we are then able to effectively mitigate for the risk of bias [35, 59], but a comprehensive and accessible registry could provide the basis that would permit a more precise understanding of how conflicts of interest relate to biases and can influence a research consensus. For example, storing conflicts of interest as computable data would provide further opportunities for researchers conducting meta-analyses, performing systematic reviews, or compiling clinical guidelines. For the purposes of analysis, such data might allow researchers to investigate the influence of conflicts of interest in more systematic ways and would dramatically reduce what is an unusually resource-intensive task in meta-research. For journalists and readers of news media, removing disclosures from behind paywalls and centralizing the information greatly simplifies the process of understanding the context in which research is undertaken and reported.

## Conclusions

While other forms of potential bias in biomedical research have been addressed through new processes and policies in the last decade, our ability to efficiently recognize and report on researcher conflicts of interest is still lagging behind other initiatives. It is imperative that we devise and support new approaches to identify, track, and account for conflicts of interest held by researchers in the biomedical sciences. This disclosure process should incorporate the key features outlined here, promoting a comprehensive and transparent approach that aims to ensure productive and trustworthy partnerships between researchers and industry.
